# Integrative Single-Cell, Spatial, and Bulk Transcriptomics Characterizes an Outcome-Associated, Spatially Organized Mesenchymal Program in *IDH*-Mutant Glioma

**DOI:** 10.3390/biology15181624

**Published:** 2026-09-15

**Authors:** Kesavi Himabindhu Vuyyuru, Vyshnavi Daggubati, Abraham Peele Karlapudi, Khurshid Ahmad

**Affiliations:** 1 Department of Bioinformatics, Vignan’s Foundation for Science, Technology and Research, Vadlamudi 522213, Andhra Pradesh, India; 2 Department of Biotechnology, Vignan’s Foundation for Science, Technology and Research, Vadlamudi 522213, Andhra Pradesh, India; 3 Department of Health Informatics, College of Applied Medical Sciences, Qassim University, Buraydah 51452, Saudi Arabia

**Keywords:** glioma, *IDH*-mutant glioma, single-cell RNA-seq, spatial transcriptomics, hypoxia, mesenchymal state, spatial null models, signature circularity, tumor microenvironment, survival analysis

## Abstract

*IDH*-mutant gliomas show variable clinical behavior that may reflect transcriptional programs organized within tumor tissue. We evaluated two curated expression scores: a differentiation-loss/Stem-NPC score (Axis1) and a mesenchymal–hypoxia score (Axis2). Public single-cell RNA-sequencing data were used to characterize malignant-cell states, and the scores were evaluated in bulk clinical cohorts using portable within-sample ranks and covariate-adjusted Cox models. Axis2 was separated into non-overlapping mesenchymal-only and hypoxia-only components. Spatial organization was tested in Visium sections from 11 patients using patient-level inference and constrained null models. The mesenchymal-only component was associated with shorter overall survival in CGGA-693 (hazard ratio per standard deviation = 1.73, 95% confidence interval 1.43–2.11) and CGGA-325 (1.41, 1.10–1.79), although molecular adjustment attenuated the association in CGGA-325. It remained spatially autocorrelated (mean Moran’s *I* = 0.297; *p* = 0.001). Removing gene overlap reduced its patient-level correlation with hypoxia from 0.798 to 0.254; residual co-organization was supported across all patients but not within either *IDH* subgroup. Axis1 showed no portable survival association. Thus, the mesenchymal component is outcome-associated and spatially organized, whereas the data do not establish hypoxia-driven organization, causality, or malignant-cell specificity.

## Simple Summary

Gliomas are brain tumors that differ widely in how aggressively they behave and in how long patients survive. We asked whether reproducible patterns of gene activity could point to clinically important features of these tumors. By combining data from individual cells, whole-tumor samples, and intact tissue sections, we identified a mesenchymal gene-expression program linked to inflammation and tissue remodeling. In two independent patient groups, this program was consistently associated with higher tumor grade and shorter survival, and within tumor tissue it was clearly organized in space. It could also be separated from the low-oxygen response that often accompanies it. A second gene-expression program, by contrast, showed no consistent relationship with survival. These findings identify a reproducible molecular feature of glioma aggressiveness and provide a basis for better biological classification and for the future development of clinically useful tumor markers.

## 1. Introduction

Diffuse gliomas are shaped by molecular genotype and by the plasticity of malignant cell states. The current World Health Organization classification treats *IDH* mutation, 1p/19q codeletion, and histological grade as the core determinants of diagnosis and prognosis [1]. Even so, tumors with the same canonical molecular labels can behave very differently, suggesting that additional transcriptional and microenvironmental programs contribute to progression risk. *IDH* mutation rewires glioma metabolism and epigenetic state [2], and longitudinal studies show that malignant gliomas continue to evolve through treatment and recurrence [3]. The central challenge is to identify malignant programs that are not only detectable in single-cell data but also portable across patient cohorts, informative about outcome, and organized in tissue.

Single-cell RNA sequencing has clarified glioma’s cellular architecture. Studies of *IDH*-mutant oligodendroglioma and astrocytoma showed that malignant cells occupy developmental and lineage-associated states rather than a single homogeneous compartment [4,5]. In glioblastoma, integrative single-cell analysis defined plastic malignant states, including astrocyte-like, oligodendrocyte-progenitor-like, neural-progenitor-like, and mesenchymal-like programs [6]. Further glioma atlases resolved the immune and stromal compartments that shape the tumor ecosystem [7,8]. The mesenchymal-like state has since proved to be inducible rather than fixed: signaling from tumor-associated macrophages can drive malignant cells toward it [9], and integrative reanalysis resolves the mesenchymal compartment into several variants, only one of which is hypoxia-associated [10]. That decomposition matters here, because it implies that a mesenchymal signature and a hypoxia signature can travel together without hypoxia being the organizing variable. *IDH*-mutant disease has been examined less often, although recent on-treatment single-cell profiling shows that *IDH*-mutant malignant states are plastic and can be shifted pharmacologically [11], and spatiotemporal multi-omic profiling has identified an immune-inflamed astrocytoma subtype with worse outcome [12]. Together, these studies provide the reference framework needed to score malignant programs in new cohorts; what remains to be established is which candidate programs are associated with outcome and how they are organized in tissue in *IDH*-mutant disease.

Spatial transcriptomics adds an anatomical layer to this problem. Recent glioma spatial studies have shown that hypoxia and regional tissue architecture can organize malignant and microenvironmental states across tumor sections [13,14], and large spatial atlases have shown that anatomical niches, necrosis, vascular regions and immune compartments can be mapped and tested statistically with spatial null models [15]. More recent work has sharpened what hypoxia does in this setting. Hypoxic regions act as a microenvironmental niche that recruits and functionally reprograms macrophages and T cells [16], and hypoxia coordinates the spatial arrangement of the myeloid compartment in a way that is associated with survival [17]; this is an effect on the microenvironment, which is not the same as hypoxia organizing a malignant program. In parallel, the spatial arrangement of transcriptional states has itself been shown to carry prognostic information beyond state abundance [18], and community single-cell and spatial atlases now provide a reference architecture for *IDH*-wildtype glioblastoma [19]. These distinctions motivate a deliberately narrow question for *IDH*-mutant glioma: is an outcome-associated mesenchymal expression program organized in tissue beyond what the tissue’s own spatial structure would produce, and is comparable organization seen in *IDH*-wildtype GBM? We do not assume that such organization defines a malignant niche, because that would require a malignant-cell specificity that spot-level data cannot provide.

We therefore built a multi-stage workflow that separates the construction of candidate scores from their independent evaluation. Established tools served as infrastructure rather than as methodological novelty: scVI provided a harmonized single-cell atlas for visualization and quality control, CellTypist supported broad cell-type annotation, non-negative matrix factorization gave an exploratory description of malignant programs, and penalized Cox modeling assessed supervised cross-cohort prognostic value. We first used public *IDH*-mutant single-cell cohorts to infer high-confidence malignant cells and to construct two curated composite scores from published reference signatures: Axis1, a differentiation-loss/Stem-NPC score, and Axis2, a mesenchymal–hypoxia score. We then evaluated these scores in *IDH*-wildtype single-cell data used as a biological contrast, in independent TCGA and CGGA bulk cohorts, and in two spatial transcriptomic datasets. Because the compact hypoxia reference set normally used as a spatial context program lies entirely within Axis2, every spatial and clinical result reported below was computed with disjoint gene sets. The evidence supports the mesenchymal component of Axis2 as a spatially organized, outcome-associated program, and places Axis1 as a separable lineage-state score that is not associated with outcome.

## 2. Materials and Methods

### 2.1. Datasets and Cohort Roles

All analyses used publicly available, de-identified human glioma data; no new specimens were generated. Each cohort was given a single, non-overlapping role so that score construction, clinical evaluation and spatial mapping remained separate. Single-cell discovery used the *IDH*-mutant glioma cohorts GSE89567 (astrocytoma, Smart-seq2) and GSE70630 (oligodendroglioma, Smart-seq2) [4,5]. Single-cell biological-contrast analyses used the *IDH*-wildtype GBM cohorts GSE131928 and GSE182109 [6,7]. Bulk clinical evaluation used TCGA-GBMLGG and the CGGA mRNAseq-693 and mRNAseq-325 cohorts [20,21,22]. Spatial evaluation used Xenium GSE302642 and Visium GSE237183 [13,14]. No sample identifier was shared between the downloaded CGGA-693 and CGGA-325 matrices (0 of 693 and 0 of 325), and the two were therefore treated as non-overlapping evaluation cohorts. *IDH* status for CGGA was taken from the recorded *IDH* mutation status; for TCGA it was assigned from the Ceccarelli pan-glioma methylation classes, with G-CIMP-high, G-CIMP-low and Codel treated as *IDH*-mutant and Classic-like, Mesenchymal-like, PA-like and LGm6-GBM as *IDH*-wildtype. A complete dataset inventory is given in Supplementary Table S1.

### 2.2. Single-Cell Preprocessing and Integration

Per-sample quality control retained cells with at least 200 and fewer than 8000 detected genes and less than 20% mitochondrial counts; genes detected in fewer than three cells were removed. Doublets were scored with Scrublet (v0.2.3) for 10x Genomics (Pleasanton, CA, USA) libraries [23]; Smart-seq2 libraries were not Scrublet-filtered because their multiplet rate is negligible. Counts were library-size normalized and log1p-transformed, and highly variable genes were selected with Scanpy (v1.11.5) [24]. A joint latent space was learned with scVI, implemented in scvi-tools (v1.4.2) [25], with source dataset as the batch covariate; Harmony (harmonypy v0.2.0) on the highly-variable-gene principal components served as a CPU fallback [26]. The integrated embedding was used only for visualization and Leiden clustering (leidenalg v0.11.0). Because a cross-platform integration is not a valid unit for differential expression, no cell-level differential-expression claim was drawn from the integrated space.

We selected highly variable genes using the Seurat flavor of the Scanpy implementation (n_top_genes = 3000), with dataset of origin as the batch key, so genes variable only within a single source were not preferentially retained. We computed principal component analysis on the scaled highly variable gene matrix with 50 components. scVI was trained with a 30-dimensional latent space, two hidden layers, a negative-binomial gene likelihood, and a maximum of 400 training epochs, with dataset of origin as the batch covariate. We also ran Harmony (maximum 30 iterations) on the same principal components as an independent check that the reported cell-type structure did not depend on a single correction method. We built the neighborhood graph in the scVI latent space with 30 neighbors, and clustered using the Leiden algorithm at resolution 0.5.

### 2.3. Cell-Type Annotation and Malignant-Cell Calling

Broad cell types were annotated with CellTypist (v1.7.1) [27] and confirmed against canonical markers. We identified malignant cells by copy-number inference with infercnvpy (v0.6.1), on the general principle that large-scale CNV signals separate malignant glioma cells from non-malignant cells [28]. CNV-neutral immune and endothelial cells served as the diploid reference. Because the infercnvpy CNV score depends on the scale of each dataset, we calibrated malignant thresholds from the reference-cell score distribution rather than fixing them a priori: a conservative high-confidence call used the reference 99th percentile, and a moderate call used the reference mean plus three standard deviations. The high-confidence calls were carried forward for all downstream analyses.

Automated annotation used CellTypist with majority voting, applying the Developing_Human_Brain and Immune_All_Low reference models; we then reconciled the automated labels with canonical marker expression at the cluster level and used the cluster-level consensus label in all downstream analyses. Copy-number inference used infercnvpy with a running window of 250 genes, a step of 10 genes and a chunk size of 3000 cells, with gene coordinates from the GENCODE v43 (GRCh38) annotation. To prevent the largest samples from dominating the diploid reference, we capped the immune and endothelial reference set at 30,000 cells by random subsampling under the fixed seed. We assigned malignant status from the resulting CNV score and annotation label, using the reference-calibrated thresholds described above.

### 2.4. Patient-Level Structure and Pseudoreplication Control

Cells were mapped to their patient or sample of origin from cohort metadata. We made quantitative comparisons at the patient or sample level, or by learning programs per patient, so individual cells were never treated as independent replicates. Patient-level axis statistics were restricted to patients with at least 20 high-confidence malignant cells. The same principle governed the spatial data, in which sections are nested within donors. The 19 Visium sections came from 11 patients: 6 *IDH*-mutant patients contributed 1 section each, whereas 5 *IDH*-wildtype patients contributed 13 sections, including repeated sections from ZH1007 (2), ZH1019 (2), ZH916 (3) and ZH881 (5). We therefore computed every Visium statistic per section, averaged within patient, and tested across the 11 patient-level values with the Wilcoxon signed-rank test; we do not report section-level *p*-values as if sections were independent. The full section-to-patient mapping is given in Supplementary Table S27. The six Xenium samples appear, from matching age, diagnosis, and 1p/19q status, to represent four individuals, but the GSE302642 metadata contain no explicit donor identifiers and the pairing cannot be confirmed; Xenium results were therefore analyzed at the sample level and interpreted descriptively.

### 2.5. IDH-Mutant Malignant Program Discovery

High-confidence malignant cells from the discovery cohorts defined the malignant compartment. Recurrent expression programs were sought by per-patient non-negative matrix factorization (NMF). For each patient with at least 30 high-confidence malignant cells, 2000 highly variable genes were selected and NMF was run at *k* = 4–7. Each raw program was summarized by its top 50 genes, and raw programs were consolidated into candidate consensus metaprograms by linking programs that shared at least 10 of those 50 genes and recurred in at least two patients. One consensus metaprogram, MP1, met these criteria in this configuration. Because the result depends on factorization rank, random seed, and merging threshold, we ran a one-factor-at-a-time sensitivity analysis over 11 unique configurations: 5 random seeds (42, 1, 7, 123 and 2024), 4 rank ranges (*k* = 3–5, 4–7, 5–8, and 6–9), and 4 merging thresholds (8, 10, 12 and 15 shared genes among the top 50), with the baseline configuration counted once. Each recovered metaprogram was compared with the baseline MP1 by Jaccard index and with the curated reference sets by gene overlap. Metaprogram discovery is reported as a descriptive, exploratory analysis; the two composite scores evaluated in this study were not derived from it. Complete enrichment and NMF sensitivity results are given in Supplementary Tables S3 and S26.

MP1 was characterized by hypergeometric over-representation analysis against curated glioma reference signatures and compact sets of Hallmark-derived processes. The background universe was the 16,913 genes expressed in the *IDH*-mutant malignant object, and *p*-values were adjusted by the Benjamini–Hochberg procedure.

Factorization used the nndsvda initialization with a maximum of 500 iterations. We removed genes whose expression reflects library composition rather than cell state (mitochondrial, ribosomal, hemoglobin, and heat-shock genes, identified by pattern matching on gene symbols) before factorization so they could not form a program. Each consensus metaprogram’s gene list included genes present in at least 25% of its contributing programs, truncated at 50 genes. Because the recovered metaprograms depend on seed, rank range, and merging threshold, none of which is determined by the data, the sensitivity analysis above is the reason both composite scores were defined from curated reference gene sets rather than from the NMF output: a reference definition does not move with these choices.

### 2.6. Reference-Program Scoring and Axis Definition

Two candidate composite scores were constructed from curated glioma reference signatures rather than from de novo metaprograms. We stress that they are curated composites, not discovered latent programs, and we refer to them as scores rather than as discovered axes. Axis1 (differentiation-loss/Stem-NPC) was the union of the Stem-Undifferentiated and Neftel NPC-like signatures. Axis2 (MES–hypoxia) was the union of the Neftel MES-like, Hypoxia, and MES signatures. One consequence of this construction is that all 15 genes of the compact Hypoxia reference set belong to the 26-gene Axis2 signature, so any correlation between Axis2 and that hypoxia set is definitional rather than empirical. To remove this circularity, Axis2 was decomposed into two disjoint components: Axis2-MES-only (11 genes: *CHI3L1*, *ANXA1*, *ANXA2*, *CD44*, *LGALS1*, *LGALS3*, *TIMP1*, *CLIC1*, *DDIT3*, *SERPINE1*, *EMP1*) and Axis2-hypoxia-only (15 genes). An independent hypoxia score was defined as the MSigDB Hallmark HYPOXIA gene set after removal of every Axis2 gene [29], leaving 186 genes with no overlap with Axis2, of which 169 and 170 were detected in CGGA-693 and CGGA-325 and 183 in the Visium data; disjointness was asserted programmatically at run time. All spatial and clinical results below are reported for these non-overlapping gene sets, with the overlapping sets shown alongside for comparison. Supplementary Tables S2 and S18 provide complete signature definitions and overlap audits. We scored differentiation and stress signatures as context. We computed single-cell scores with Scanpy score_genes using seed 42. We assessed score separability at the patient level using per-patient mean scores with 1000-iteration bootstrap confidence intervals, because cells within a patient are not independent observations; a cell-level correlation between scores would be pseudoreplicated and is not reported. Portable axis signatures were exported for cross-cohort use.

We computed single-cell and spot-level signature scores with the Scanpy score_genes function, which subtracts the mean expression of a control gene set sampled to match the signature genes on binned average expression; the control set comprised 50 genes per expression bin, drawn under the fixed seed. We scored a signature only when at least three of its genes were detected in the object; otherwise, we recorded it as missing rather than zero. For the targeted Xenium panel, the control-set size was scaled to the signature as min(50, max(10, 5 × number of genes)), because a fixed control set of 50 genes would be a large fraction of the panel and would absorb the signal being measured. The disjoint gene sets were defined by construction rather than by selection: Axis2-MES-only by removing every compact-hypoxia gene from Axis2, and the independent hypoxia score by removing every Axis2 gene from the Hallmark set. The run-time disjointness assertion halts the analysis, rather than silently reintroducing overlaps, if either input signature changes.

### 2.7. Bulk Clinical Evaluation

Bulk expression matrices were log-transformed. Per-gene z-scores standardized within one cohort depend on that cohort’s platform and case mix and do not transfer, so the primary scoring method was a within-sample rank score: all genes are ranked within each sample, and the signature score is the mean within-sample percentile rank of its member genes. This depends only on the ordering of genes inside a single sample, and no training-cohort mean or standard deviation is carried across platforms. Cohort z-scores were retained as a sensitivity analysis, and the two are reported side by side. Signature gene recovery was tabulated per cohort. Association with tumor grade was tested by Spearman correlation and the Kruskal–Wallis test, across all samples and within *IDH*-mutant samples. Association with overall survival was tested with Cox proportional hazards models in lifelines (v0.30.3) [30]. Every fitted model is reported with its cohort, analysis scope, molecular subtype, number of eligible patients, complete-case sample size, number of deaths, percentage of patients dropped for missing covariates, hazard ratio per standard deviation with 95% confidence interval, *p*-value, Harrell’s concordance index, and Schoenfeld proportional-hazards tests for the global model and for the score term; covariate missingness is tabulated for every field used. Kaplan–Meier curves were estimated from a median split and compared by the log-rank test. Survival analyses were performed within *IDH*-mutant patients in each cohort.

Treatment and molecular covariates were handled as follows. CGGA records radiotherapy status, chemotherapy status, and primary/recurrent/secondary type, so treatment-adjusted models included age, grade, radiotherapy, chemotherapy, and recurrence. No harmonized surgery or extent-of-resection variable was available in the downloaded clinical matrices, so none could be included; this remains a possible source of residual confounding. The treatment fields are incomplete (radiotherapy missing in 6.8% of CGGA-693 and 4.6% of CGGA-325; chemotherapy missing in 6.6% and 6.5%), so complete-case sample sizes and death counts are reported for every adjusted model, and we do not claim that treatment confounding has been eliminated. Molecular subtype was assigned by a prespecified WHO-2021 rule before any survival model was fitted: *IDH*-mutant with 1p/19q codeletion was classed as oligodendroglioma, *IDH*-mutant without codeletion as astrocytoma, and cases of unknown codeletion status were excluded rather than pooled into an ambiguous group. Subtype-stratified models are prespecified secondary analyses and are underpowered, particularly in the codeleted stratum. To ask whether bulk associations could be driven by non-malignant tissue content, ESTIMATE stromal and immune signature scores were computed [31] and added as covariates in a composition-adjusted sensitivity model; these two 141-gene signatures share no gene with Axis2. We report a composition adjustment rather than a calibrated absolute purity percentage, because the ESTIMATE purity formula is calibrated to that package’s own score scale. Bulk expression cannot establish malignant-cell origin, and this analysis is a sensitivity check, not evidence of malignant-cell specificity. As a further sensitivity analysis, the clinical baseline was extended with *MGMT* promoter methylation, 1p/19q codeletion and histologic class, all of which are recorded in the CGGA clinical matrices; they are absent from the downloaded TCGA GBMLGG Xena clinical matrix, so this extended model is restricted to CGGA. *MGMT* status is missing for 21.8% of CGGA-693 and 5.8% of CGGA-325, so complete-case fitting removes patients. To separate attenuation caused by adjustment from attenuation caused by that loss, the treatment-adjusted model was also refitted on exactly the patients available to the extended model, giving three comparable estimates per score and cohort (Supplementary Table S29). Indicator variables that become constant within a stratum, such as 1p/19q inside the codeleted stratum, were dropped, and each such drop is recorded per model in Supplementary Table S21.

Within-sample ranks were computed once per cohort across all genes within each sample, so the rank score depends only on the ordering of genes within a patient. Cox models were fitted without penalization on complete cases after excluding patients with non-positive follow-up time, and no model was fitted when fewer than 25 patients or fewer than 10 events remained. We standardized the score within each model’s complete-case set, so each hazard ratio is per standard deviation of the score among the patients analyzed. Proportional hazards were assessed with scaled Schoenfeld residuals under a rank time transform, and both the global and the score-specific *p*-values are reported in Supplementary Table S21. ESTIMATE stromal and immune scores were computed in Python from the published signature gene lists [31] rather than through the original R package, because only the signature scores are used as covariates and the package’s calibrated absolute purity percentage is not.

### 2.8. Cross-Cohort Machine-Learning Survival Model

To quantify whether Axis2 adds prognostic information beyond standard clinical variables, a supervised regularized Cox proportional hazards model was trained in *IDH*-mutant CGGA-693 patients and evaluated without refitting in TCGA-GBMLGG and CGGA-325. Two nested feature sets were compared: a clinical baseline of age plus grade, and the same baseline plus the Axis2 score. The model used elastic-net regularization (penalizer 0.1, L1 ratio 0.5) in lifelines, and discrimination was quantified by Harrell’s concordance index. Separately from these fixed cross-cohort point estimates, within-cohort stability was examined in CGGA-693 and CGGA-325 with a treatment-adjusted baseline of age, grade, radiotherapy, chemotherapy, and recurrence status. In each of 2000 patient-level resamples, we fitted the baseline and baseline-plus-score models on the same resample and evaluated them on the same out-of-bag patients; we recorded the paired difference in concordance and report its median and 2.5th–97.5th percentile interval. This resampling analysis is distinct from the fixed age-plus-grade external point estimates and is read as within-cohort stability, not as a confidence interval for those estimates. No grade-classification target was reported, because grade is itself a clinical covariate and such a classifier would be circular.

Because the baseline and baseline-plus-score models are fitted on the same bootstrap sample and evaluated on the same out-of-bag patients, the resulting interval is an interval on the paired difference in concordance, not a comparison of two independently estimated concordances; it is the latter that inflates apparent gains in cross-cohort comparisons. Within the bootstrap, both models were fitted with a ridge penalty of 0.1 for numerical stability in small resamples. A resample was discarded if it left fewer than 20 out-of-bag patients, fewer than 10 events in the training portion, or fewer than 5 events in the out-of-bag portion, and the number of resamples actually used is reported with every estimate (Supplementary Table S25).

### 2.9. Spatial Analysis

Spatial datasets were scored with the same portable axis and context signatures. In Xenium, the 541-gene custom panel reduces Axis2 to its MES core plus *HILPDA*, the only hypoxia gene on the panel, and reduces Axis1 to four genes; this limitation is stated with every Xenium result. Visium provided full-transcriptome coverage of both axes and of the context programs. We quantified spatial autocorrelation as Moran’s *I* on a squidpy (v1.8.1) spatial-neighbor graph [32]. Because the compact hypoxia context set is a subset of Axis2, every spatial analysis was recomputed with the disjoint Axis2-MES-only score and the independent Hallmark hypoxia score defined in Section 2.6; the circular and non-circular results are reported together so that the size of the definitional component is visible. We averaged section-level statistics within each patient before testing. The spot-group contrast divided the spots or cells of each sample into high (top quartile) and low (bottom quartile) groups for the score under analysis and quantified each context feature by Cliff’s delta, with Visium stratified by *IDH* class and Xenium by tumor or peritumoral role. These are contrasts between groups of spots, not niches: spot-level data cannot show that the difference originates in malignant cells.

Free within-sample label randomization does not preserve the observed spatial autocorrelation and can therefore give an anti-conservative benchmark when both variables are spatially structured. Two spatially constrained nulls were used instead. Moran spectral randomization (singleton method [33]) was the primary constrained null: the doubly centered, row-normalized spatial weights matrix was eigendecomposed, the observed score was projected onto its eigenvectors, and the signs of the spectral coefficients were randomized. This preserves the observed Moran’s *I* exactly, together with the sample mean and variance. Equal-capacity spatial block reassignment was the secondary null: spots were partitioned into compact blocks of equal size and whole blocks were moved to randomly chosen positions. This is an exact permutation of the observed value vector, although local structure is retained only approximately, because source and destination blocks can differ in shape. Across the 19 sections, block surrogates retained a median 0.58 of the observed Moran’s *I* (interquartile range 0.48–0.65). On average, 96.5% of spots changed position in the all-spot analyses and 89.6% in the lineage-depleted analyses; spots held fixed in incomplete blocks were 0.1% of a section on average and at most 0.7%. A toroidal shift was not used, because irregular tissue boundaries make wraparound invalid. The test statistic was the difference in mean neighboring independent-hypoxia score between spots in the top and bottom quartiles of the Axis2-MES-only score. Section-level *p*-values were adjusted by Benjamini–Hochberg separately within each subset and null across the 19 sections; both nominal *p*-values and *q*-values are reported in Supplementary Table S20. Section counts are descriptive, because several sections come from the same patient. Formal inference is at the patient level: each section was converted to *z* = (T_obs − mean_null)/SD_null against its spectral-randomization null, each patient was summarized by the mean *z* over its sections, and the patient vector was tested with the Wilcoxon signed-rank test. With 6 *IDH*-mutant and 5 *IDH*-wildtype patients, the subgroup analyses are exploratory and underpowered.

Visium spots were retained at a minimum of 500 counts and 200 detected genes, and Xenium cells at a minimum of 10 counts. The spatial neighborhood graph was built in squidpy with a generic coordinate type and six nearest neighbors for Visium, matching the hexagonal lattice, and 15 nearest neighbors for Xenium; weights were row-standardized before use, so that Moran’s *I* and the neighbor statistic are not driven by differences in neighbor count at tissue edges. The test statistic was defined across neighboring spots rather than within a spot for a specific reason: a same-spot correlation would remain partly definitional even with disjoint gene sets, because both scores are measured from the same transcript pool, and averaging the hypoxia score over a spot’s neighbors before the contrast removes that route. Under every null, only the Axis2-MES-only map was randomized; the independent hypoxia map was held fixed throughout, so the null distribution reflects rearrangement of the program being tested and not of the feature against which it is tested. Sections with fewer than 150 spots, or with fewer than 10 spots in either quartile group, were prespecified as not testable; no section met either criterion, and all 19 sections were analyzed.

For spectral randomization, the score vector was centered, expanded on the eigenvectors of the doubly centered, symmetrized weights matrix, sign-randomized coefficient by coefficient, then reconstructed and rescaled to the observed mean and standard deviation. The full eigenvector basis was retained rather than truncated, so reconstruction is exact and each surrogate carries the observed Moran’s *I* by construction. For block reassignment, the block size was chosen per section from the range 45–75 spots so as to strand the fewest spots in the incomplete remainder block; a single fixed size of 60 spots would have left up to 12.3% of spots held fixed in the smallest section, which is enough to weaken the null. Each section was evaluated against 1000 surrogates for the block-reassignment null (and for the unconstrained label-randomization benchmark retained in Supplementary Tables S12–S15) and 500 for spectral randomization, whose eigendecomposition is the limiting cost. All permutation *p*-values are two-sided and use the add-one correction, (number of surrogates at least as extreme in absolute value + 1)/(number of surrogates + 1), so that no *p*-value is reported as exactly zero. We recomputed the histological region gradient (Section 3.5) with the Axis2-MES-only score and aggregated it from section to patient before any statistics were taken; we assessed the monotonic trend as a per-patient Spearman correlation across ordered regions for patients contributing at least three regions. Only four *IDH*-wildtype patients carry region annotations, and this analysis is therefore reported as exploratory (Supplementary Table S28).

#### Non-Malignant-Lineage-Depletion Sensitivity Analysis

Visium spots are 55 micrometers across and contain mixtures of malignant and non-malignant cells, so a spot-level association cannot on its own be attributed to malignant cells. Matched single-cell reference profiles were not available for these sections, so reference-based deconvolution was not possible; we therefore implemented an explicitly defined non-malignant-lineage-depletion sensitivity analysis. Non-malignant lineage panels (myeloid, T cell, oligodendrocyte, endothelial, pericyte and neuron) were scored after every Axis2 and independent-hypoxia gene had been removed from them, so that the enrichment criterion could not be correlated by construction with the score being tested. We z-scored panel scores within each section and averaged them into a single non-malignant composite, then repeated all spatial tests on the bottom tertile of that composite, which retained 33.4% of spots. This procedure reduces but does not remove non-malignant contribution, and we do not claim malignant-cell specificity on this basis.

We computed the tertile cut within each section so spot retention does not depend on differences in overall composition between sections, and we recomputed every spatial statistic and null model unchanged on the restricted spot set. This is a depletion-of-normal-lineage argument, not evidence of malignant origin, which spot-level expression cannot establish.

### 2.10. Exploratory Candidate Vulnerability Nomination

To place Axis2 in a translational context without making causal target-discovery claims, Axis2 genes were cross-referenced with public DepMap 24Q4 CRISPR gene-effect data and DGIdb 5.0 drug–gene interaction annotations [34,35]. DepMap models were filtered on the metadata terms glioma, glioblastoma, astrocytoma, oligodendroglioma, brain, central nervous system, and CNS. For each Axis2 gene, the mean and minimum CRISPR gene-effect scores were summarized across the glioma/CNS-matched models, together with the fraction of models below the dependency thresholds of −0.5 and −1.0. DGIdb served only as a drug–gene annotation resource; we did not interpret interactions as clinically actionable. Because this analysis is peripheral to the main claims and cannot distinguish glioma-selective dependency from broad essentiality, we report it in the Supplementary Material and summarize it in Section 4. It is hypothesis-generating and was not used as evidence of glioma-selective drug targets.

### 2.11. Statistical Analysis and Software

Group comparisons used the Mann–Whitney U or Kruskal–Wallis test; correlations used Pearson and Spearman coefficients; survival used Cox proportional hazards models and the log-rank test. Patient-level comparisons of spatial statistics used the Wilcoxon signed-rank test. Multiple testing was controlled by the Benjamini–Hochberg false discovery rate wherever many sets or features were tested [36]. Confidence intervals for per-patient axis means were estimated by a 1000-iteration nonparametric bootstrap; confidence intervals for the within-cohort change in concordance index used a 2000-iteration paired patient-level bootstrap with out-of-bag evaluation. The equal-capacity block reassignment and Moran spectral randomization nulls were implemented in numpy and scipy following Wagner and Dray [33]. A fixed random seed of 42 was used throughout. Analyses were performed in Python 3.11.15 using numpy 2.4.3, pandas 2.3.3, scipy 1.17.1, Scanpy 1.11.5, anndata 0.12.11, squidpy 1.8.1, scvi-tools 1.4.2 (PyTorch 2.10.0), harmonypy 0.2.0, leidenalg 0.11.0, CellTypist 1.7.1, infercnvpy 0.6.1, Scrublet 0.2.3, scikit-learn 1.8.0, statsmodels 0.14.6, lifelines 0.30.3, gseapy 1.2.1, matplotlib 3.10.9 and seaborn 0.13.2. R 4.5.3 was available for cross-checking. Supplementary Table S16 lists software versions.

The fixed seed of 42 was applied to every stochastic step, including integration, clustering, factorization, signature control-set selection, surrogate generation, and bootstrap resampling. All tests were two-sided. Confidence intervals for hazard ratios are Wald intervals from the Cox fit, and intervals for bootstrap quantities are percentile intervals. Where a result is reported for a subgroup small enough that the test has a non-trivial minimum attainable *p*-value, that minimum is stated alongside the result.

## 3. Results

### 3.1. Integrated Single-Cell Atlas Identifies Malignant IDH-Mutant Glioma Programs

**Figure 1:**
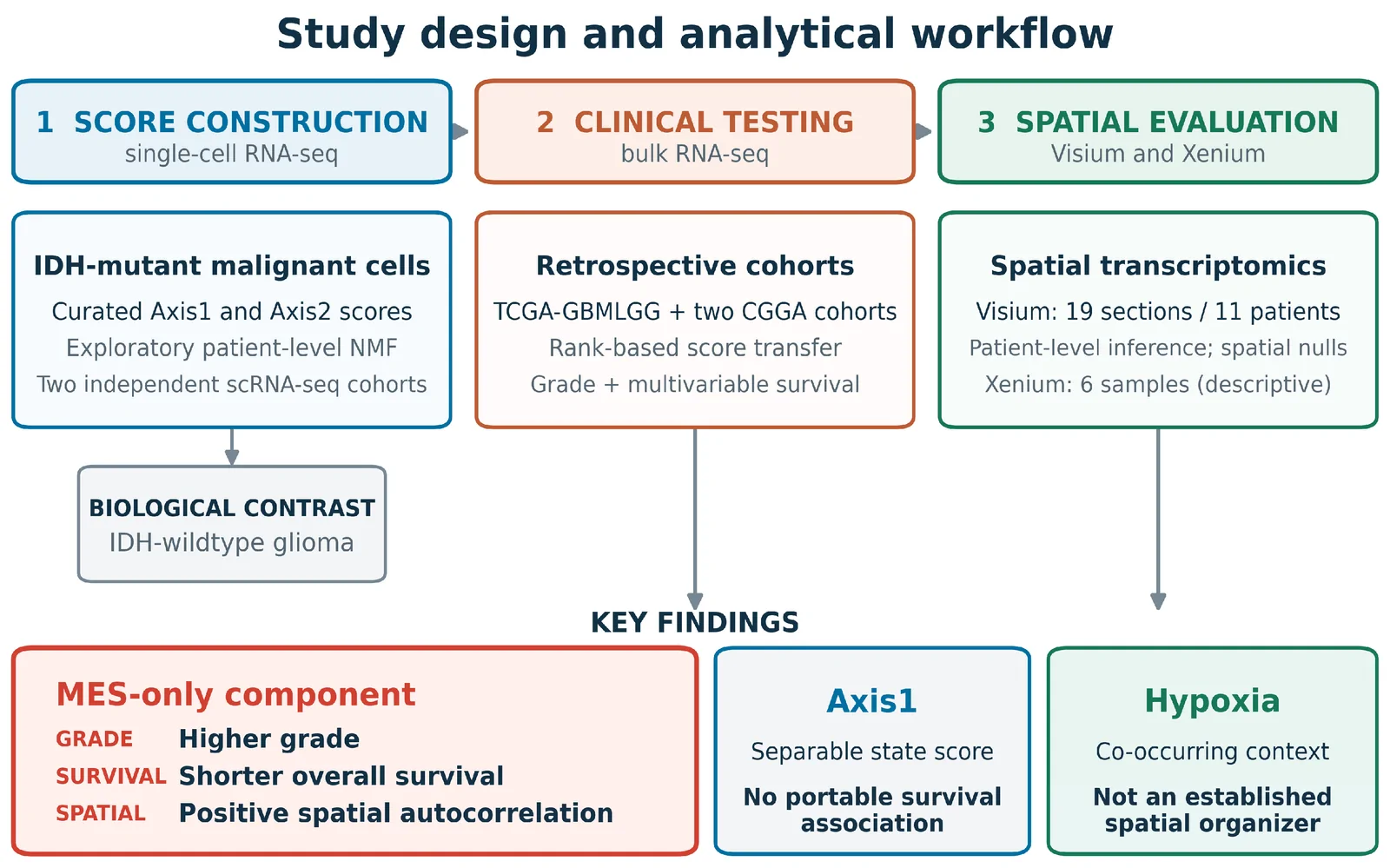
Study design and analytical workflow. Two curated reference-signature scores (Axis1 and Axis2) were characterized in *IDH*-mutant malignant single-cell data; patient-level NMF was exploratory and did not define the scores. *IDH*-wildtype datasets served as biological contrasts, and the TCGA-GBMLGG/CGGA cohorts and Visium/Xenium datasets provided clinical and spatial evaluation, respectively. The mesenchymal-only component of Axis2 was associated with higher grade and shorter overall survival and showed positive spatial autocorrelation. Axis1 showed no portable survival association, and hypoxia was a co-occurring context rather than an established spatial organizer.

Following the three-stage design of discovery, clinical evaluation and spatial mapping summarized in Figure 1, we assembled an integrated single-cell atlas of *IDH*-mutant glioma from two deep Smart-seq2 cohorts, GSE89567 and GSE70630, and isolated the malignant compartment by copy-number inference. This yielded 1122 high-confidence malignant cells from 13 patients (572 astrocytoma and 550 oligodendroglioma). The joint scVI embedding was used for visualization and Leiden clustering only; all downstream expression programs were learned per patient.

Within this malignant compartment, the canonical glioma reference programs were recovered with near-complete gene coverage, including the Hypoxia, MES, Proliferation, astrocyte-like, oligodendrocyte-like and Neftel mesenchymal-like signatures. Per-patient lineage composition spanned the canonical astrocyte-like, oligodendrocyte-like, stem/undifferentiated, and cycling states, which provided the lineage backdrop against which candidate expression programs were evaluated.

Per-patient NMF followed by cross-patient consolidation identified one consensus metaprogram, MP1. In the baseline configuration, MP1 was dominated by stress-response genes, with secondary mesenchymal- and hypoxia-associated features, including *VIM*, *DDIT3*, *DDIT4*, *ALDOC* and *JUN*/*FOS*. Over-representation analysis confirmed enrichment for immediate-early/NF-kappaB stress-response genes, astrocytic/glial and heat-shock/unfolded-protein programs and, secondarily, mesenchymal-EMT and hypoxia/glycolysis genes. MP1 showed no over-representation of stem/undifferentiated, NPC-like, OPC-like or cycling programs. The per-patient strategy limits pseudoreplication and batch-driven structure, but a sensitivity sweep showed that MP1 was not robustly recoverable: only seven patients met the inclusion threshold, one or two metaprograms were recovered across the eleven configurations, and the Jaccard similarity of the leading program to the frozen MP1 reference had a median of 0.370 (range 0.250–0.887). Across configurations, overlap ranged from 2 to 3 of the 26 Axis2 genes, 1 to 7 of the 11 stress/immediate-early genes, and 3 to 6 of the 18 astrocyte-like genes; in the published configuration, the overlaps were 3/26, 7/11, and 6/18. MP1 therefore does not specifically motivate a mesenchymal–hypoxia axis and is reported as exploratory. The composite scores evaluated below were constructed independently from published reference signatures.

### 3.2. Construction of Two Candidate Malignant Scores: Differentiation Loss and MES–Hypoxia

Because the metaprogram analysis was unstable and did not resemble a mesenchymal–hypoxia program, the two candidate scores were constructed independently of it, from curated glioma reference gene sets chosen for portability across bulk and spatial platforms. Axis1 (differentiation-loss/Stem-NPC; 18 genes, including *SOX4*, *SOX11*, *DCX* and *STMN2*) captures a neurodevelopmental stem/progenitor-like state. Axis2 (MES–hypoxia; 26 genes, including *CD44*, *VIM* and the hypoxia core genes *HILPDA*, *VEGFA*, *LDHA*, *SLC2A1* and *NDRG1*) captures a mesenchymal–hypoxic state. The discovery object contained 1122 high-confidence malignant cells. Because cells within a patient are not independent observations, we assessed separability at the patient level rather than the cell level. Across the nine patients who met the scoring threshold, the two scores showed no statistically supported correlation (Pearson *r* = 0.318, 95% CI −0.543 to 0.784, *p* = 0.404; Spearman rho = 0.183, 95% CI −0.621 to 0.842, *p* = 0.637), although with only nine patients the uncertainty is substantial. We therefore describe the two scores as separable and do not claim that they are orthogonal or statistically independent. Pooling the 1054 cells of these nine patients into a single cell-level correlation (Pearson *r* = −0.013, *p* = 0.67) would be pseudoreplicated and is not evidence of independence. Patient-level correlation results are given in Supplementary Table S4.

**Figure 2:**
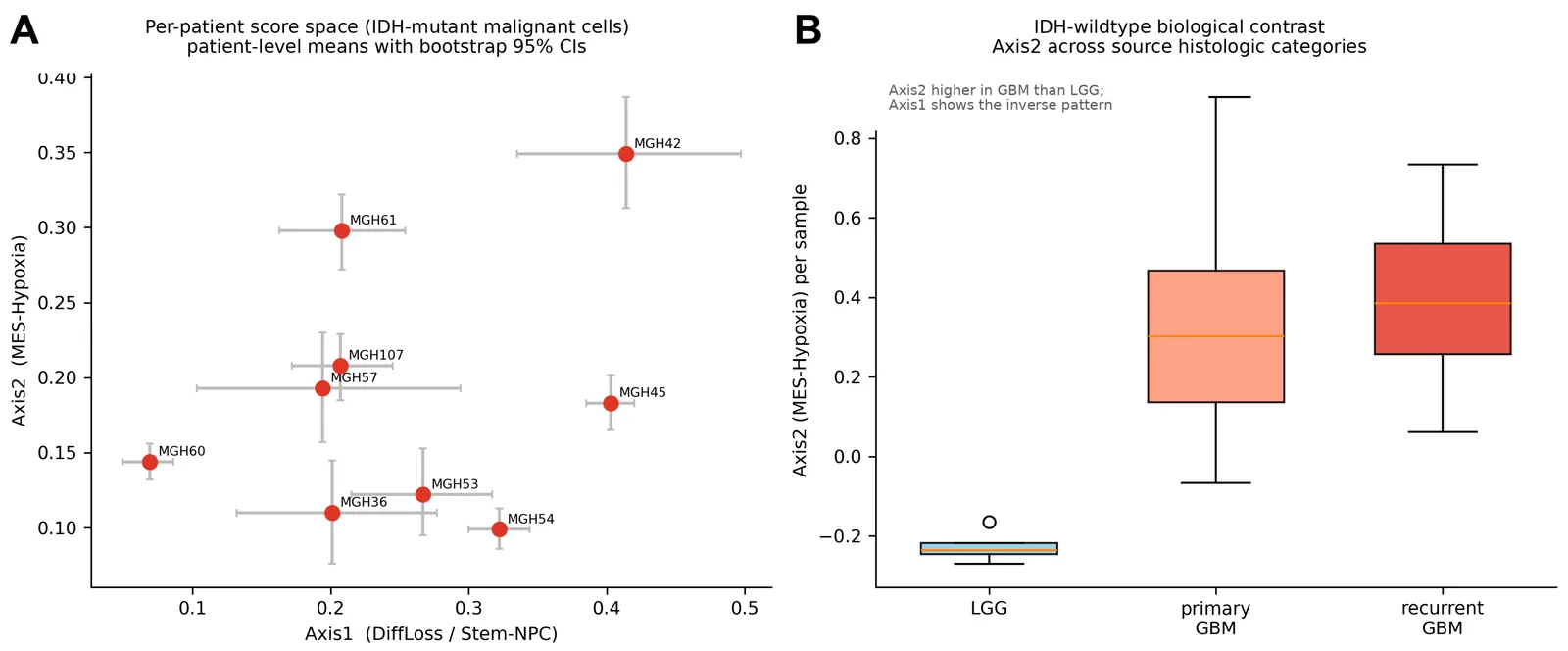
Construction of two candidate malignant scores in *IDH*-mutant glioma. (**A**) Per-patient axis space for *IDH*-mutant malignant cells. Each point is one patient, positioned by its mean Axis1 and Axis2 scores; error bars are bootstrap 95% confidence intervals. The two scores are separable at the patient level, but with only nine evaluable patients, the correlation is imprecisely estimated (Pearson *r* = 0.318, 95% CI −0.543 to 0.784) and independence is not claimed. (**B**) *IDH*-wildtype biological contrast: Axis2 scores increase from LGG to primary and recurrent GBM, while Axis1 shows the inverse trend. Both axes are curated reference signatures, not NMF-derived programs.

The separability was also visible in individual tumors, which occupied distinct regions of the Axis1–Axis2 plane (Figure 2A): MGH45 was Axis1-high but Axis2-low, MGH61 was comparatively Axis2-high, and MGH42 scored high on both. At this sample size, however, a patient’s position on one score says little about its position on the other, and the panel is descriptive.

To see how the scores behave across an independent biological contrast, we evaluated both in an *IDH*-wildtype cohort spanning source-labeled lower-grade glioma and GBM samples; this comparison was not treated as a model of *IDH*-mutant progression. Axis2 rose sharply from LGG to primary and recurrent GBM, whereas Axis1 moved in the opposite direction (Figure 2B). *IDH*-wildtype tumors are a different disease entity, not a later stage of *IDH*-mutant glioma, so this contrast motivates the score but does not validate it. The findings separated a lineage-state score from a MES–hypoxia score and nominated Axis2, rather than Axis1, for clinical evaluation. Detailed biological-contrast results are given in Supplementary Table S5.

### 3.3. The Mesenchymal Component of Axis2 Carries the Association with Grade and Survival

We next scored both axes in three bulk RNA-seq cohorts: TCGA-GBMLGG (*n* = 694), CGGA-693 (*n* = 693) and CGGA-325 (*n* = 325). The downloaded CGGA matrices shared no sample identifiers and were treated as non-overlapping evaluation cohorts. Under the platform-portable within-sample rank score, Axis2 was associated with histological grade within *IDH*-mutant tumors in both CGGA cohorts (Spearman rho = 0.281, *p* = 7.6 × 10^−8^, *n* = 355 in CGGA-693; rho = 0.484, *p* = 1.3 × 10^−11^, *n* = 174 in CGGA-325). Decomposing Axis2 into its disjoint components showed that this association is carried by the mesenchymal genes: Axis2-MES-only reached rho = 0.301 and 0.488, whereas Axis2-hypoxia-only reached only rho = 0.132 (*p* = 0.013) and 0.271. The same ordering held under cohort z-scoring (MES-only rho = 0.281 and 0.515 versus hypoxia-only 0.098 and 0.291). The grade association of Axis1 was weak and cohort-dependent (rho = 0.035, *p* = 0.51 in CGGA-693; 0.242, *p* = 1.3 × 10^−3^ in CGGA-325), consistent with its secondary status. Detailed grade-association results are given in Supplementary Table S23.

In treatment-adjusted Cox models within *IDH*-mutant patients, adjusted for age, grade, radiotherapy, chemotherapy and primary/recurrent status, the mesenchymal component was associated with worse overall survival in both CGGA cohorts (HR per SD = 1.73, 95% CI 1.43–2.11, *p* = 3.4 × 10^−8^, *n* = 320 with 148 deaths in CGGA-693; HR = 1.41, 95% CI 1.10–1.79, *p* = 5.7 × 10^−3^, *n* = 159 with 91 deaths in CGGA-325). Complete-case analysis dropped 10.1% and 9.1% of eligible patients; missingness is tabulated in Supplementary Table S22. The proportional-hazards assumption held for the score term (Schoenfeld *p* = 0.996 and 0.356), although the global test was significant in CGGA-325 (*p* = 0.005) and in the molecularly extended models in CGGA-693 (*p* = 0.021) and CGGA-325 (*p* = 0.040). The score hazard ratios should therefore be read as average associations over follow-up, and time-varying effects of other covariates remain a limitation. The hypoxia-only component was weaker and was not significant in CGGA-325 (HR = 1.18, 95% CI 0.96–1.45, *p* = 0.11). Because the treatment fields are incomplete, treatment confounding cannot be excluded. Under the prespecified WHO-2021 subtype rule, the association was present in *IDH*-mutant astrocytoma in CGGA-693 (HR = 1.39, 95% CI 1.11–1.75, *p* = 4.2 × 10^−3^, *n* = 183 with 106 deaths) but not in CGGA-325 (HR = 1.17, 95% CI 0.88–1.56, *p* = 0.26, *n* = 100 with 73 deaths); the codeleted strata had only 32 and 16 deaths. Extending the baseline with *MGMT* promoter methylation, 1p/19q codeletion and histologic class separated the cohorts: the association persisted in CGGA-693 (HR = 1.56, 95% CI 1.24–1.96, *p* = 1.3 × 10^−4^, *n* = 228 with 115 deaths) but not in CGGA-325 (HR = 1.25, 95% CI 0.97–1.62, *p* = 0.091, *n* = 143 with 83 deaths). A same-patient refit in CGGA-325 remained associated (HR = 1.44, 95% CI 1.11–1.86, *p* = 5.4 × 10^−3^), which attributes the attenuation to the molecular and histologic adjustment rather than to complete-case attrition. *MGMT* promoter methylation and 1p/19q codeletion were unavailable in the downloaded TCGA clinical matrix. Supplementary Tables S21 and S29 provide complete Cox-model outputs and molecular-adjustment analyses.

Because bulk expression mixes malignant with non-malignant tissue, we asked whether these associations could reflect composition. The mesenchymal component correlated strongly with the ESTIMATE immune and stromal scores (Spearman rho = 0.78 and 0.71 in CGGA-693; 0.73 and 0.73 in CGGA-325), even though those two 141-gene signatures share no genes with Axis2. In a cohort-z-score, composition-adjusted sensitivity analysis, adding the stromal and immune scores left the mesenchymal component associated with survival in both cohorts (HR = 1.57, 95% CI 1.13–2.19, *p* = 7.2 × 10^−3^ in CGGA-693; HR = 1.84, 95% CI 1.27–2.67, *p* = 1.3 × 10^−3^ in CGGA-325), whereas the hypoxia-only component was not (*p* = 0.12 and 0.050) and the independent Hallmark hypoxia score lost significance in CGGA-693 (*p* = 0.12). This cohort-z-score model is a sensitivity analysis and is distinct from the portable within-sample rank scoring used in the primary models. Bulk expression cannot establish malignant-cell origin, and residual composition confounding remains possible. Supplementary Table S24 provides detailed composition analyses.

To assess the added value of supervised learning, we trained an elastic-net Cox model on *IDH*-mutant CGGA-693 and evaluated it without refitting in the independent cohorts. In a single train/test analysis, adding Axis2 to an age-plus-grade baseline raised the concordance index from 0.674 to 0.725 in CGGA-693, from 0.589 to 0.667 in held-out TCGA-GBMLGG, and from 0.720 to 0.772 in held-out CGGA-325. These fixed external point estimates have no confidence intervals in the current analysis. In a separate within-cohort paired out-of-bag bootstrap with treatment-adjusted baselines (age, grade, radiotherapy, chemotherapy and recurrence), the median change was +0.014 (95% interval −0.022 to +0.044) in CGGA-693 and +0.019 (−0.012 to +0.053) in CGGA-325. These intervals measure within-cohort stability under treatment-adjusted baselines; they are not confidence intervals for the fixed external point estimates. Across all 20 cohort-by-score configurations, no interval excluded zero (Supplementary Figure S2 and Supplementary Table S25). We therefore do not claim a demonstrated improvement in discrimination: the observed changes were small and statistically uncertain.

**Figure 3:**
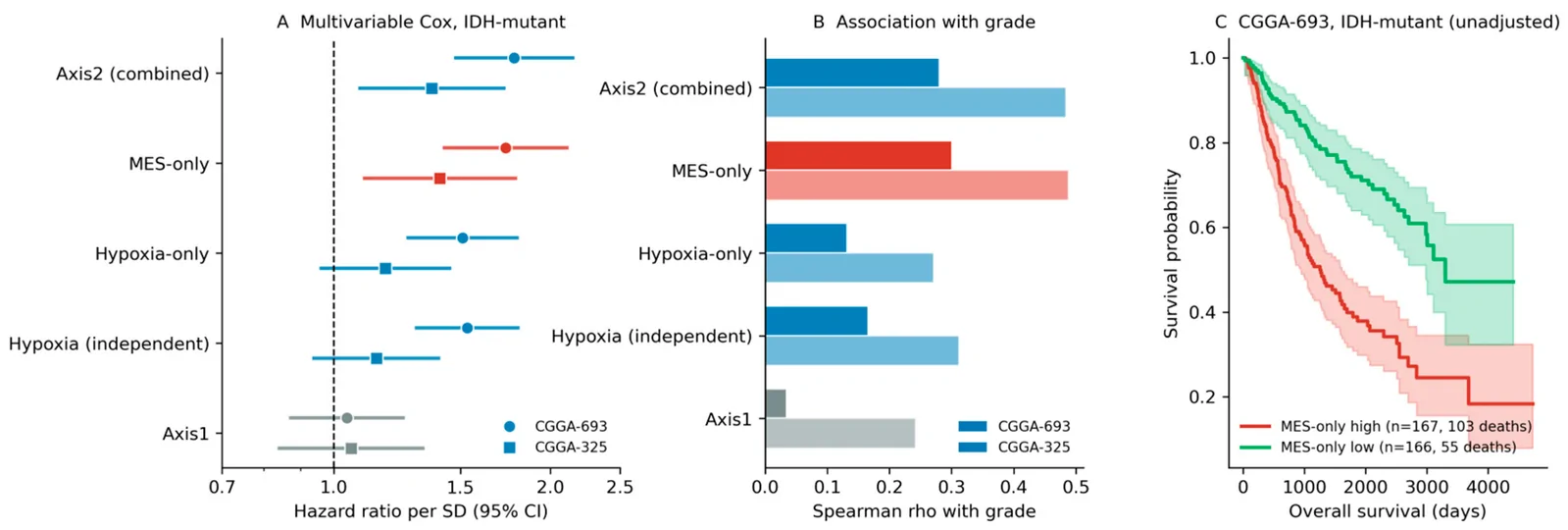
Clinical evaluation of Axis2 in *IDH*-mutant glioma. (**A**) Adjusted hazard ratios per SD (95% CIs) for Axis2 and its components in CGGA-693 and CGGA-325. Models included age, grade, radiotherapy, chemotherapy, and primary/recurrent status. (**B**) Spearman correlations between each score and tumor grade. All analyses used within-sample rank scores. (**C**) Unadjusted Kaplan–Meier analysis of the mesenchymal-only score in CGGA-693 (median split; *n* = 333, 158 deaths; log-rank *p* = 7.3 × 10^−10^). In (**A**,**B**), colour identifies the score (red, mesenchymal-only; grey, Axis1; blue, the remaining scores) and is used for emphasis only; the cohort is given by marker shape in (**A**) and by bar shade in (**B**), as indicated in the legends. The dashed vertical line in (**A**) marks a hazard ratio of 1. Shaded bands in (**C**) are 95% confidence intervals. Complete model specifications, sample sizes, and sensitivity analyses are given in the Supplementary Material.

Axis1, by contrast, showed no reproducible independent association with survival. Under the platform-portable rank score its effect collapsed in both CGGA cohorts (HR per SD = 1.04, 95% CI 0.87–1.25, *p* = 0.64 in CGGA-693; HR = 1.06, 95% CI 0.84–1.33, *p* = 0.63 in CGGA-325), even though the same models with cohort z-scores gave nominally significant hazard ratios of 1.23 (*p* = 0.028) and 1.36 (*p* = 0.014). The Axis1 association was thus not robust to the scoring method and was not regarded as a portable biological association. Its apparent protective effect in the pooled TCGA analysis disappeared after adjustment for *IDH* status and grade, which identifies it as an *IDH*-confounded signal rather than an independent risk axis. These results support the mesenchymal component of Axis2, and not Axis1, as the outcome-associated score taken forward to spatial analysis. Figure 3 summarizes the clinical associations, and Supplementary Figure S1 presents the complete Kaplan–Meier grid.

### 3.4. Removing Signature Circularity Attenuates the Axis2-Hypoxia Coupling but Not the Spatial Organization

**Figure 4:**
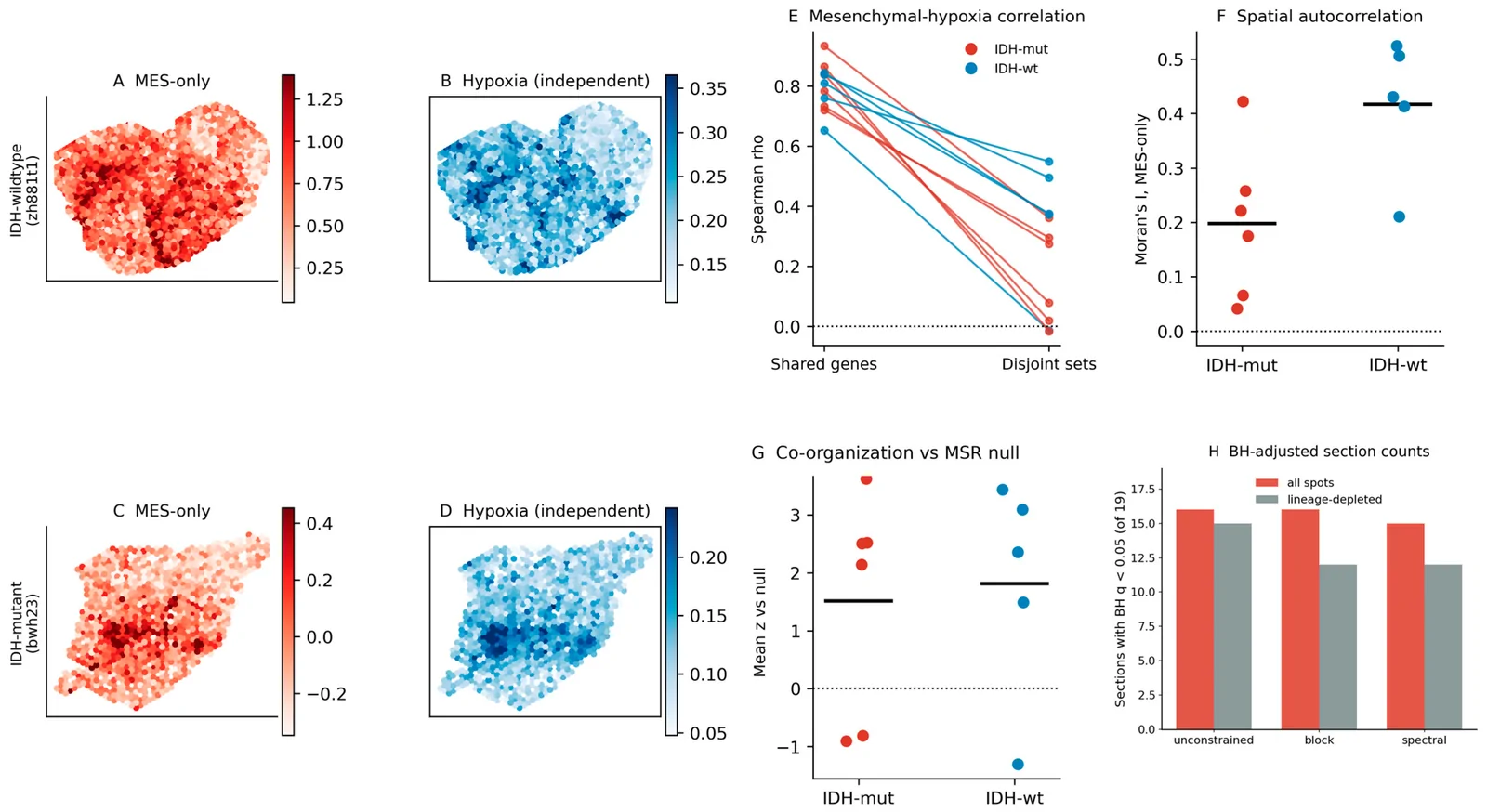
Spatial organization of the mesenchymal component. (**A**–**D**) Representative Visium maps of the mesenchymal-only and non-overlapping Hallmark hypoxia scores in *IDH*-wildtype and *IDH*-mutant sections. (**E**) The patient-level correlation decreased from 0.798 with overlapping scores to 0.254 with disjoint scores. (**F**) Patient-level spatial autocorrelation of the mesenchymal-only score (mean Moran’s *I* = 0.297). (**G**) Standardized effects against the MSR null were significant across all patients (mean *z* = 1.65; *p* = 0.014) but not within either *IDH* subgroup. (**H**) Descriptive numbers of BH-significant sections under the unconstrained, block, and MSR nulls: 16, 16, and 15 for all spots and 15, 12, and 12 for lineage-depleted spots, respectively. Formal inference is patient-level, as in (**G**).

To test whether Axis2 is spatially organized, we scored it in full-transcriptome Visium data comprising 19 sections from 11 patients (Figure 4). With the compact hypoxia reference set, Axis2 appeared tightly coupled to hypoxia in every sample, with a patient-level mean Spearman rho of 0.798. That result is definitional rather than empirical: all 15 genes of that hypoxia set belong to the 26-gene Axis2 signature, so the comparison is between a set and its own superset. Repeating the analysis with the disjoint Axis2-MES-only component and a Hallmark hypoxia score containing no Axis2 gene reduced the patient-level correlation from 0.798 to 0.254, a decrease of approximately 68% in mean rho on removal of the shared genes. In the six *IDH*-mutant patients, the residual coupling was not statistically supported (mean rho = 0.169, Wilcoxon *p* = 0.063); in the five *IDH*-wildtype patients, it was larger but likewise did not reach significance at that sample size (mean rho = 0.356, *p* = 0.125). After excluding shared genes, the coupling between the mesenchymal program and hypoxia is neither near-universal nor, within *IDH*-mutant tumors, statistically supported. Spatial organization itself survived the correction: the mesenchymal-only component, which contains no hypoxia gene, remained significantly spatially autocorrelated across patients (mean Moran’s *I* = 0.297, range 0.042–0.524, Wilcoxon *p* = 0.0010 across 11 patients), including within the *IDH*-mutant subset alone (mean I = 0.197, *p* = 0.031), with stronger clustering in *IDH*-wildtype (mean I = 0.417) than in *IDH*-mutant tumors. Per-section and patient-level non-circular spatial results are given in Supplementary Table S19.

We next asked whether the local co-organization of the mesenchymal component and independent hypoxia exceeded what the tissue’s existing spatial structure would produce. Because free label randomization can overstate co-organization, Moran spectral randomization (MSR) was the primary null, and equal-capacity spatial block reassignment was the secondary null. The 19 sections come from 11 patients, so section-level counts are descriptive and formal inference is at the patient level. Each section was standardized against its own MSR null, and each patient contributed the mean of its sections. Across the 11 patients, the effect was positive (mean *z* = 1.65) and significant by the Wilcoxon signed-rank test (*p* = 0.014). After Benjamini–Hochberg adjustment within each subset and null, 15 of 19 sections had *q* < 0.05 under MSR, 16 under block reassignment, and 16 under unconstrained permutation. Restricting the analysis to the bottom tertile of a six-lineage non-malignant score retained 33.4% of spots and gave a similar patient-level result (mean *z* = 1.36, *p* = 0.019); the corresponding FDR-significant section counts were 12, 12 and 15. Neither *IDH* subgroup was significant on its own (*IDH*-mutant *p* = 0.156, *n* = 6; *IDH*-wildtype *p* = 0.125, *n* = 5). The depletion analysis reduces but does not remove non-malignant contribution and does not positively identify malignant spots. Without matched single-cell reference deconvolution, malignant-cell specificity cannot be demonstrated, and we therefore speak of a mesenchymal expression program rather than of a malignant program or niche. Complete constrained-null results are presented in Supplementary Figure S3 and Supplementary Table S20.

Single-cell-resolution Xenium data from six *IDH*-mutant samples showed a descriptively similar pattern. From their matching clinical attributes, the samples appear to represent four individuals, but donor identity cannot be confirmed from the available metadata. Axis2 was higher in tumor cores than in peritumoral tissue (mean 0.64 versus 0.28) and was spatially clustered (Moran’s *I* 0.14–0.48). We therefore made core/peritumoral comparisons at the sample level and did not interpret them as confirmed paired analyses. The Xenium panel is targeted and reduces Axis2 to its mesenchymal core plus *HILPDA* as the only hypoxia gene, so Xenium corroborates cellular-scale MES organization but cannot test the hypoxia coupling. Sample-level Xenium results are given in Supplementary Table S11.

### 3.5. Exploratory Regional and Vascular Patterns in IDH-Wildtype GBM

Within the *IDH*-wildtype samples that carry histological region annotations, we asked whether the mesenchymal program varies systematically from the infiltrating margin to the tumor core. Scored with the mesenchymal-only gene set, which excludes the hypoxia genes, and aggregated from section to patient so that repeated sections of one tumor count once, the mean score rose from the infiltrating margin (0.32, four patients) through T1 contrast-enhancing tissue (0.74, three patients) to bulk tumor (0.94, two patients) but did not continue to rise in the necrotic core (0.78), which is represented by a single patient. Only two patients contributed three or more annotated regions, so no patient-level trend test is possible. These data do not support a monotonic edge-to-core gradient culminating in the necrotic core; we report only that the mesenchymal score is lower at the infiltrating margin than in enhancing or bulk tumor in the small number of annotated *IDH*-wildtype patients available. The observation is exploratory. Patient-aware regional summaries are given in Supplementary Table S28.

The inverse association between the mesenchymal score and vascular features was not universal. Across the 13 *IDH*-wildtype sections, neighbor-level vascular associations were heterogeneous (mean Cliff’s delta = −0.21): eight effects were negative overall, and seven of the eight T1-enhancing, bulk or necrotic sections showed negative effects, whereas three of the four infiltrating-margin sections showed positive effects. These unconstrained-permutation section-level results are descriptive, because they do not preserve spatial autocorrelation and the 13 sections come from only five patients. The same pattern was not consistent in the *IDH*-mutant Visium samples or in Xenium. We therefore describe exploratory regional and vascular patterns in selected high-grade *IDH*-wildtype compartments, not a general property of Axis2.

**Figure 5:**
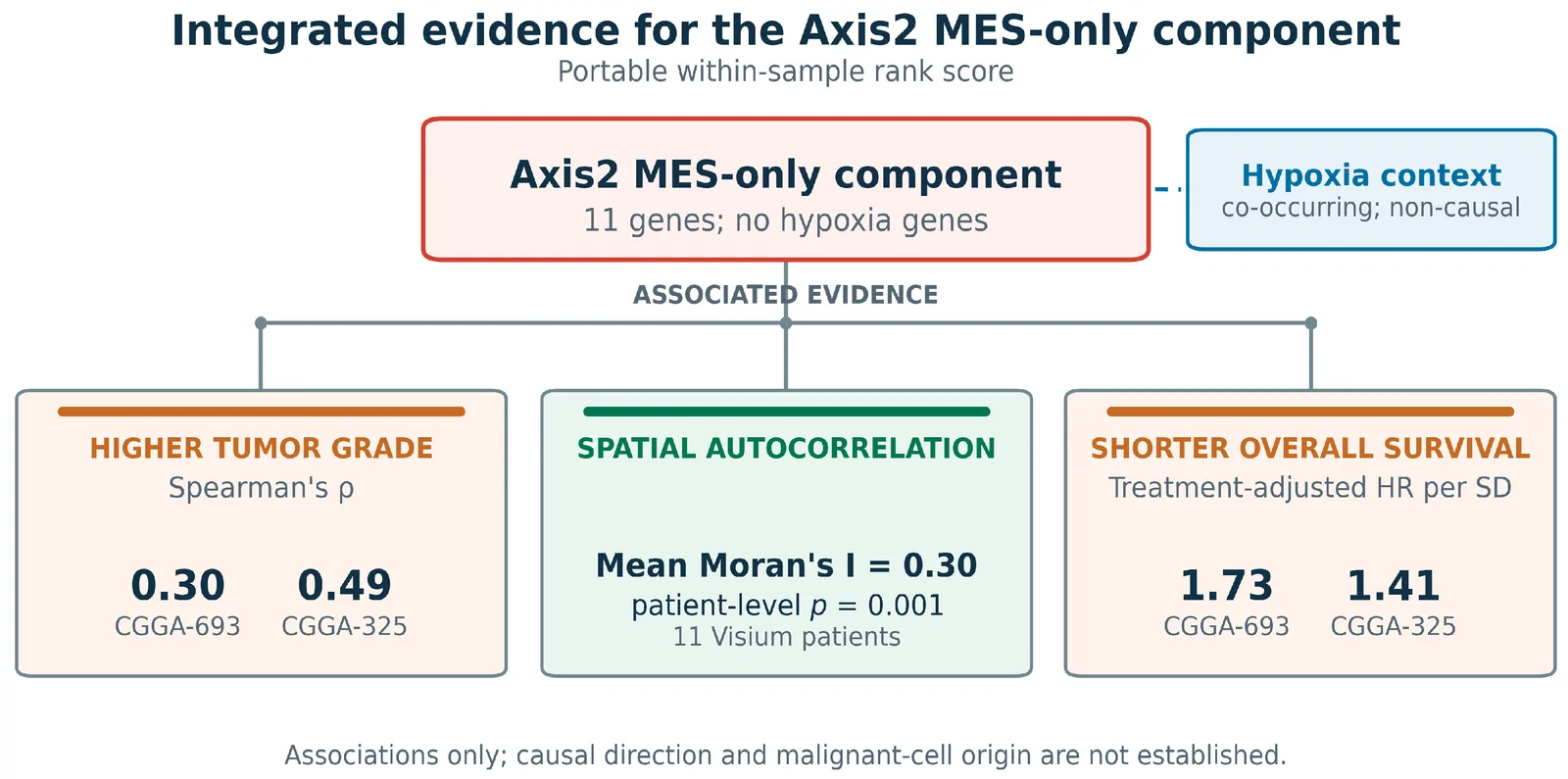
Integrated model of the Axis2 MES-only component. The 11-gene score contains no hypoxia gene. In CGGA-693 and CGGA-325, it was associated with higher grade (Spearman’s ρ = 0.30 and 0.49, respectively) and shorter overall survival (treatment-adjusted HR per SD = 1.73 and 1.41, respectively). It was also spatially autocorrelated in Visium (mean Moran’s *I* = 0.30; patient-level *p* = 0.001; *n* = 11). Hypoxia is shown as a co-occurring context; no causal relationship is inferred. All links denote association; causality and malignant-cell origin are not established.

Taken together, these results support the model summarized in Figure 5. The mesenchymal component of a curated mesenchymal–hypoxia composite score is associated with grade and overall survival in two non-overlapping retrospective cohorts, survives adjustment for the available treatment fields and for stromal/immune composition, and is spatially organized in tissue. Its coupling to hypoxia, however, was largely definitional: once disjoint gene sets and spatially constrained null models are used, the residual coupling is supported across patients as a whole but reaches significance in neither *IDH* subgroup separately, and the available patient numbers cannot resolve whether it differs between *IDH* classes. In high-grade *IDH*-wildtype GBM, the program also showed exploratory regional and vascular associations. Axis1 is separable but is not associated with outcome under portable scoring, and is retained only as a descriptive lineage-state score.

## 4. Discussion

This study evaluates a curated mesenchymal–hypoxia composite score, Axis2, and finds that its mesenchymal component is the most consistently outcome-associated and spatially organized signal across single-cell, bulk clinical and spatial glioma datasets. The analysis uses established AI/ML tools as infrastructure rather than proposing a new algorithm: scVI organized the single-cell atlas, CellTypist supported annotation, NMF gave an exploratory description of malignant programs, and an elastic-net Cox model tested supervised cross-cohort prognostic value. Starting from *IDH*-mutant malignant cells, we constructed two candidate scores from published reference signatures, a differentiation-loss/Stem-NPC score and a mesenchymal–hypoxia score. At the patient level the two were separable but imprecisely correlated (Pearson *r* = 0.318, 95% CI −0.543 to 0.784, *n* = 9), so we treat them as distinct without claiming independence. In two non-overlapping clinical cohorts, the mesenchymal component was associated with higher grade and worse survival within *IDH*-mutant tumors after adjustment for age, grade, radiotherapy, chemotherapy and recurrence, and after further adjustment for stromal and immune composition. Axis1 remained biologically interpretable but showed no portable association with outcome.

The findings fit the established glioma cell-state frameworks and add a clinically and spatially grounded layer to them. Earlier single-cell studies described malignant glioma states spanning astrocytic, oligodendrocytic, neural-progenitor and mesenchymal-like programs [4,5,6]. Our de novo *IDH*-mutant analysis recovered an astrocytic/stress metaprogram in the baseline configuration and showed little proliferative activity, consistent with the relatively indolent biology of the available *IDH*-mutant discovery samples. A systematic sensitivity analysis shows that metaprogram to be sensitive to seed and rank, with a median Jaccard overlap of only 0.370 against MP1 across the 11 configurations, and to resemble a stress/astrocytic rather than a mesenchymal–hypoxia program in every configuration (at most 3 of the 26 Axis2 genes). It therefore does not specifically motivate a mesenchymal–hypoxia axis. The two-axis framework was accordingly defined from curated glioma reference signatures rather than by treating the limited de novo metaprogram structure as exhaustive, and Axis2 should be read as a constructed and evaluated composite score, not as a discovered program.

Axis2 was the most consistently outcome-associated of the scores tested, and decomposition localized that association to its mesenchymal genes. It increased with grade in both CGGA cohorts and was associated with worse overall survival in *IDH*-mutant tumors after adjustment for age, grade, radiotherapy, chemotherapy and primary/recurrent status. Because bulk expression mixes malignant and non-malignant tissue, we asked whether this could reflect tumor composition: the mesenchymal component correlates strongly with the ESTIMATE immune and stromal scores (Spearman rho 0.78 and 0.71 in CGGA-693; 0.73 and 0.73 in CGGA-325) despite sharing no gene with them. With those scores added as covariates, the mesenchymal component remained associated with survival (HR = 1.57, *p* = 7.2 × 10^−3^ in CGGA-693; HR = 1.84, *p* = 1.3 × 10^−3^ in CGGA-325), whereas the hypoxia-only component did not (*p* = 0.12 and 0.050) and the independent Hallmark hypoxia score lost significance in CGGA-693 (*p* = 0.12). This separation of the mesenchymal from the hypoxic component, seen independently in the grade analysis, the composition-adjusted survival analysis and the portable-scoring analysis, is the clearest positive result of the study. It remains an association in retrospective cohorts, and bulk data cannot establish malignant-cell origin. The supervised elastic-net analysis, once evaluated by a paired patient-level bootstrap with out-of-bag evaluation, gave a consistent but small improvement in concordance whose confidence interval includes zero (+0.014, 95% CI −0.022 to +0.044 in CGGA-693), so we do not present it as demonstrated added predictive value. TCGA supported the grade association but was weaker for *IDH*-mutant survival, which may reflect fewer events and greater clinical heterogeneity; we retain this as a cohort-level nuance rather than suppress it.

The spatial analysis is where the choice of gene sets and of null model matters most. Axis2 appears almost universally coupled to hypoxia across Visium sections, but the compact hypoxia reference set ordinarily used for that comparison lies entirely within Axis2, so the correlation is in large part definitional. Recomputing it with disjoint gene sets reduced the patient-level correlation from 0.798 to 0.254 and left it unsupported within the six *IDH*-mutant patients (*p* = 0.063). Likewise, within-sample label permutation does not preserve spatial autocorrelation and so cannot provide a spatially matched null for testing specific co-organization. Under Moran spectral randomization, with equal-capacity block reassignment as a secondary null, the standardized effect was positive across the eleven patients as a whole (mean *z* = 1.65, Wilcoxon *p* = 0.014; non-malignant-lineage-depleted spots, mean *z* = 1.36, *p* = 0.019), but neither *IDH* subgroup reached significance on its own (*IDH*-mutant *p* = 0.156, *n* = 6; *IDH*-wildtype *p* = 0.125, *n* = 5). We therefore make no subtype-specific claim; in particular, with five patients the *IDH*-wildtype subgroup cannot attain a two-sided exact Wilcoxon *p* < 0.05. What does survive is the spatial organization of the mesenchymal component itself, which remains significantly autocorrelated at the patient level (mean Moran’s *I* = 0.297, *p* = 0.0010) with a gene set that contains no hypoxia gene. The supportable claim is therefore that the mesenchymal expression program shows positive spatial autocorrelation in both *IDH* classes, with formal support in the pooled analysis and in the *IDH*-mutant subset. Residual coupling to hypoxia was supported only in the pooled patient-level analysis, not within either subgroup, and the regional and vascular patterns in high-grade *IDH*-wildtype GBM remain descriptive.

The working biological model must therefore be stated with care. A mesenchymal expression program is spatially organized in glioma tissue and is associated with higher grade and worse outcome. In *IDH*-wildtype GBM it showed a positive descriptive association with hypoxia and was lower at the infiltrating margin than in enhancing or bulk tumor, although with only four region-annotated patients this spatial trend is exploratory and we do not describe it as a monotonic edge-to-core gradient. In *IDH*-mutant tumors, the present data do not support a hypoxia-organized interpretation once circularity and spatial structure are properly controlled. We therefore do not propose that hypoxia establishes or maintains this state; an observational design cannot distinguish hypoxia driving the mesenchymal program, the mesenchymal program arising in tissue that is already hypoxic, or both reflecting a shared regional determinant. This more restricted reading agrees with independent work. Integrative reanalysis of glioblastoma resolves the mesenchymal compartment into several variants, only one of which is hypoxia-associated [10], so a mesenchymal and a hypoxia signature can co-occur without hypoxia being the organizing variable. Where hypoxia has been shown to coordinate spatial organization in glioma, the organized compartment is the myeloid and immune microenvironment rather than a malignant program [16,17]. Our data are compatible with that picture, and the strong correlation of the mesenchymal component with the ESTIMATE immune and stromal scores is consistent with a substantial microenvironmental contribution that bulk and spot-level expression cannot separate from malignant-cell signal. Independently, the finding that spatial arrangement carries prognostic information beyond state abundance [18] supports treating spatial organization as a property worth testing in its own right, which is what the present analysis does.

An exploratory cross-reference with public DepMap CRISPR-dependency data and DGIdb annotations, summarized in Supplementary Figure S4 and Supplementary Table S17, identified *ALDOA* and *PGK1* as the only Axis2 genes that met the prespecified dependency threshold and returned a drug–gene annotation. Both are core glycolytic enzymes and were frequently dependent among the 91 glioma/CNS-matched models. Because no pan-cancer comparator was used, this analysis does not establish glioma-selective dependency and does not support clinical target nomination.

Several limitations should be noted. The discovery component uses public datasets with few *IDH*-mutant malignant patients: only seven patients entered metaprogram discovery and only nine were evaluable for the patient-level score comparison, so those analyses are descriptive and underpowered. Axis1 and Axis2 are curated composites assembled from published reference signatures; they are not newly discovered latent programs, and Axis2 in particular is internally heterogeneous, which is why its mesenchymal and hypoxic components are reported separately throughout. The bulk cohorts are retrospective, the treatment covariates are incomplete (6–7% missing), no harmonized surgery/extent-of-resection variable was available, and composition adjustment reduces but cannot remove the contribution of non-malignant tissue; bulk expression cannot establish malignant-cell origin. Subtype-stratified models are underpowered, particularly in the 1p/19q-codeleted stratum (32 and 16 deaths). The survival association is also not uniformly robust to molecular adjustment: once *MGMT* methylation, 1p/19q codeletion and histologic class are added to the baseline, the mesenchymal association persists in CGGA-693 but falls below significance in CGGA-325, whereas on the same patients without those covariates it does not, so the loss in CGGA-325 is attributable to the covariates rather than to the smaller sample. We therefore regard the clinical association as replicated in one cohort and as not independent of standard molecular markers in the other. The concordance gain from adding Axis2 is small, and its confidence interval includes zero. In the spatial data, the 19 Visium sections come from only eleven patients, and the six Xenium samples appear to represent four individuals but lack explicit donor identifiers in the GEO metadata; malignant-cell specificity was approximated by restricting to non-malignant-lineage-depleted spots rather than by matched single-cell reference deconvolution, which was not available for these sections. Xenium remains panel-limited and cannot test hypoxia coupling. *IDH*-wildtype GBM is a distinct disease entity and was used here as a biological contrast, not as a model of *IDH*-mutant progression. All spatial and clinical analyses are observational and cannot establish causation. Finally, no wet-lab perturbation or histology-based experimental validation was performed.

Within these limits, the convergence of bulk clinical evaluation, composition- and treatment-adjusted survival models, platform-portable scoring and spatially constrained null-model testing supports a mesenchymal expression program as a replicated, spatially organized and outcome-associated feature of glioma, subject to the covariate sensitivity reported above. The same analyses show what does not survive scrutiny: the hypoxia coupling in *IDH*-mutant tumors is largely a consequence of overlapping gene sets, the de novo metaprogram is unstable and biologically distinct from the axis, and the machine-learning gain in discrimination is not statistically established. We report both outcomes because the negative results are as informative as the positive one: they identify signature circularity and spatially unconstrained null models as concrete sources of overstatement in spatial-transcriptomic niche analysis, and they localize the replicated signal to the mesenchymal rather than the hypoxic component of the program.

## 5. Conclusions

This integrative analysis identifies the mesenchymal-only component of a curated mesenchymal–hypoxia score as a spatially organized glioma feature associated with higher tumor grade and shorter overall survival in two retrospective cohorts. The survival association remained after adjustment for treatment and composition, although additional molecular covariates attenuated it in one cohort. Patient-level spatial analyses further showed non-random organization of the mesenchymal program across tumor sections. Removing the overlapping genes markedly weakened its correlation with hypoxia, indicating that the observed signal is principally mesenchymal rather than evidence of hypoxia-driven organization. Axis1 remained biologically distinct but showed no consistent association with survival. These computational findings support the use of portable within-sample rank scoring, non-overlapping signature definitions and patient-level spatial inference for evaluating clinically relevant glioma expression programs, and they prioritize the mesenchymal component for independent experimental and clinical validation.

## Data Availability

All datasets analyzed in this study are publicly available. Single-cell datasets were obtained from GEO accessions GSE89567, GSE70630, GSE131928, and GSE182109. Bulk cohort data were obtained from TCGA-GBMLGG through UCSC Xena and from CGGA mRNAseq-693 and mRNAseq-325. Spatial datasets were obtained from GEO accessions GSE302642 and GSE237183. Public dependency and drug–gene annotation resources were obtained from DepMap 24Q4 Public and DGIdb 5.0. The analytical results reported in the manuscript are documented in Supplementary Tables S1–S29. Detailed model specifications, software versions, and processed result tables are provided in the same tables. The analysis scripts are available from the corresponding author upon reasonable request.

## Supplementary Materials

The following supporting information can be downloaded at: https://www.mdpi.com/article/10.3390/biology15181624/s1, The Supplementary Material comprises Supplementary Figures S1–S4 and Supplementary Tables S1–S29. Tables S1–S17 provide dataset inventory, signatures, enrichment analyses, clinical models, sensitivity analyses, spatial tests, software versions and exploratory dependency annotation. Tables S12–S15 report unconstrained-permutation analyses, which are descriptive and are not used as inferential evidence. Figure S1 presents the full *IDH*-mutant Kaplan–Meier grid; Figure S2 presents the within-cohort treatment-adjusted paired out-of-bag bootstrap, in which none of the 20 intervals excluded zero; Figure S3 presents non-circular spatial analyses, constrained nulls and patient-level inference; and Figure S4 presents exploratory DepMap/DGIdb annotation. Tables S18–S29 contain signature definitions and overlap audits (S18), circular and disjoint Visium summaries (S19), constrained spatial nulls with nominal *p* and BH *q* values (S20), full Cox reporting including proportional-hazards tests (S21), missingness (S22), grade analyses (S23), cohort-z-score composition sensitivity models (S24), within-cohort treatment-adjusted paired bootstrap results distinct from the fixed external point estimates (S25), NMF sensitivity (S26), section-to-patient mapping (S27), patient-aware regional analysis (S28) and molecular adjustment with a same-patient refit (S29). The supplementary tables reproduce the model-level results quoted in the manuscript.
